# Effects of glucagon like peptide-1 to mediate glycemic effects of weight loss surgery

**DOI:** 10.1007/s11154-014-9291-y

**Published:** 2014-06-21

**Authors:** Marzieh Salehi, David A. D’Alessio

**Affiliations:** 1 Department of Internal Medicine, Division of Endocrinology, Diabetes, & Metabolism, University of Cincinnati College of Medicine, 260 Stetson, Suite 4200, Cincinnati, OH 45219-0547 USA; 2 Cincinnati VA Medical Center, Cincinnati, OH USA

**Keywords:** Gastric bypass surgery, Sleeve gastrectomy, GLP-1, Hyperinsulinemia, Diabetes, Hypoglycemia

## Abstract

To date, weight loss surgeries are the most effective treatment for obesity and glycemic control in patients with type 2 diabetes. Roux-en-Y gastric bypass surgery (RYGB) and sleeve gastrectomy (SG), two widely used bariatric procedures for the treatment of obesity, induce diabetes remission independent of weight loss while glucose improvement after adjustable gastric banding (AGB) is proportional to the amount of weight loss. The immediate, weight-loss independent glycemic effect of gastric bypass has been attributed to postprandial hyperinsulinemia and an enhanced incretin effect. The rapid passage of nutrients into the intestine likely accounts for significantly enhanced glucagon like-peptide 1 (GLP-1) secretion, and postprandial hyperinsulinemia after GB is typically attributed to the combined effects of elevated glucose and GLP-1. For this review we focus on the beneficial effects of the three most commonly performed bariatric procedures, RYGB, SG, and AGB, on glucose metabolism and diabetes remission. Central to this discussion will be the extent to which the effects of surgery are mediated by GLP-1. Better understanding of these mechanisms could provide insight to development of novel therapeutic strategies for treatment of diabetes as well as refinement of surgical techniques.

## The clinical role of weight-loss surgeries

Bariatric surgeries were originally categorized based on what was commonly believed to be their mechanisms of action, volume restriction, malabsorption or both. Malabsorptive procedures, such as biliopancreatic diversion and jejunoileal bypass, are reconfigurations of the small intestine with the intent of diminishing the area of intestinal mucosa available for nutrient absorption. Procedures termed restrictive, such as vertical banded gastroplasty or laparoscopic adjustable gastric banding (AGB), limit the capacity of the stomach to accommodate food with an expected secondary restriction of calorie intake. Among bariatric surgeries, Roux-en-Y gastric bypass (RYGB), including components of restriction (a small gastric pouch) and malabsorption (bypass of the stomach and proximal portion of small intestine), was endorsed by National Institutes of Health Consensus Development Panel as the ‘gold standard’ procedure in 1991 because of its predictable high weight-loss efficacy and low post-operative complication rates [1]. Subsequently, this procedure has been the most commonly performed bariatric surgery (60–80 %) in the US, with more than 700,000 persons in the US over the last decade having undergone RYGB [2]. In recent years sleeve gastrectomy (SG), a procedure in which there is selective removal of the gastric fundus and greater curvature of the stomach without intestinal bypass, has also become popular, with comparable weight loss to RYGB [3], from a technically easier procedure; SG comprised approximately one-third of bariatric procedures in 2012 [4]. While bariatric surgical procedures are still categorized as restrictive or malabsorptive, this dichotomization has been questioned recently [5] and there is certainly more to be learned about the mechanisms of action of RYBG and SG.

While there are only limited data on the long-term results of bariatric surgery, these have been generally positive. The Swedish Obese Subjects trial compared groups of more than 2,000 obese subjects with bariatric surgery and a similar number with nonoperative treatment of obesity for 10 years. In this cohort surgical treatment of obesity reduced mortality, incident diabetes, and rates of myocardial infarction, stroke and cancer [6]. In addition, short-term observational trials have shown that weight loss following bariatric surgery is associated with a reduction in key obesity-related metabolic comorbidities, such as type 2 diabetes, hypertension, and dyslipidemia [7]. Interestingly, some metabolic benefits of bariatric surgery do not seem to be dependent on weight loss, occurring soon after surgery before significant changes in body weight. Notable among these are improvements in diabetes, with better glycemic control and a need for less diabetes treatment frequently observed in the immediate postoperative period [8].

Recovery from diabetes following bariatric surgeries has been well documented. In a prospective, longitudinal study based on bariatric-specific data from 28,616 obese diabetic patients, rate of remission or improvement in diabetes at 1 year after RYGB was 83 % compared to 55 % and 44 % after SG and AGB, with BMI reductions of ~15 kg/m^2^ compared to 12 and 7 kg/m^2^, respectively [7]. In keeping with these observational studies, three randomized clinical trials have recently demonstrated that a greater portion of patients with uncontrolled diabetes achieve target A1C levels at 1–2 years after RYGB compared to those receiving lifestyle and/or medical interventions alone [9–11]. Compared to AGB, RYGB is more effective in inducing remission of metabolic conditions such as diabetes, hypertension and dyslipidemia. This is in keeping with greater effectiveness in weight loss as reported from a recent prospective multi-central study of 2,348 patients with type 2 diabetes in the US [12]. Preliminary evidence suggests that RYGB may be more effective for treating diabetes than SG [13] with longer-lasting effects even when weight reduction is comparable [14].

## Weight-loss independent glycemic effect of bariatric surgeries

It is well recognized that weight loss whether induced by life style interventions or a bariatric surgery improves glucose tolerance, mainly by enhancing insulin sensitivity and improving fasting glucose kinetics [15–17]. Therefore, some of the profound reduction in mean blood glucose among persons with type 2 diabetes who have bariatric procedures can be attributed to surgical-induced weight loss. In fact, improvement in glucose homeostasis with gastric restrictive surgeries, similar to life-style interventions, occurs over weeks to months in parallel with, and proportional to, the amount of weight loss [18]. However, much of the effect of RYGB and, to lesser degree, SG, is immediate and partly independent of the amount of weight loss [17, 19–21].

In a longitudinal prospective study of subjects with type 2 diabetes and recent RYGB, the need for antidiabetic medications was eliminated in more than 30 % at the time of discharge from hospital [22]. The likelihood of diabetes remission was inversely related to the duration of diabetes suggesting that factors related to shorter exposure to hyperglycemia, e.g. healthier islet β-cells, respond better to the surgical treatment. This immediate, weight-loss independent glucose-lowering effect of RYGB has been attributed to alterations in postprandial glucose homeostasis as a result of enhanced nutrient fluxes [23, 24] and enteroinsular signaling [25, 26].

**Fig. 1:**
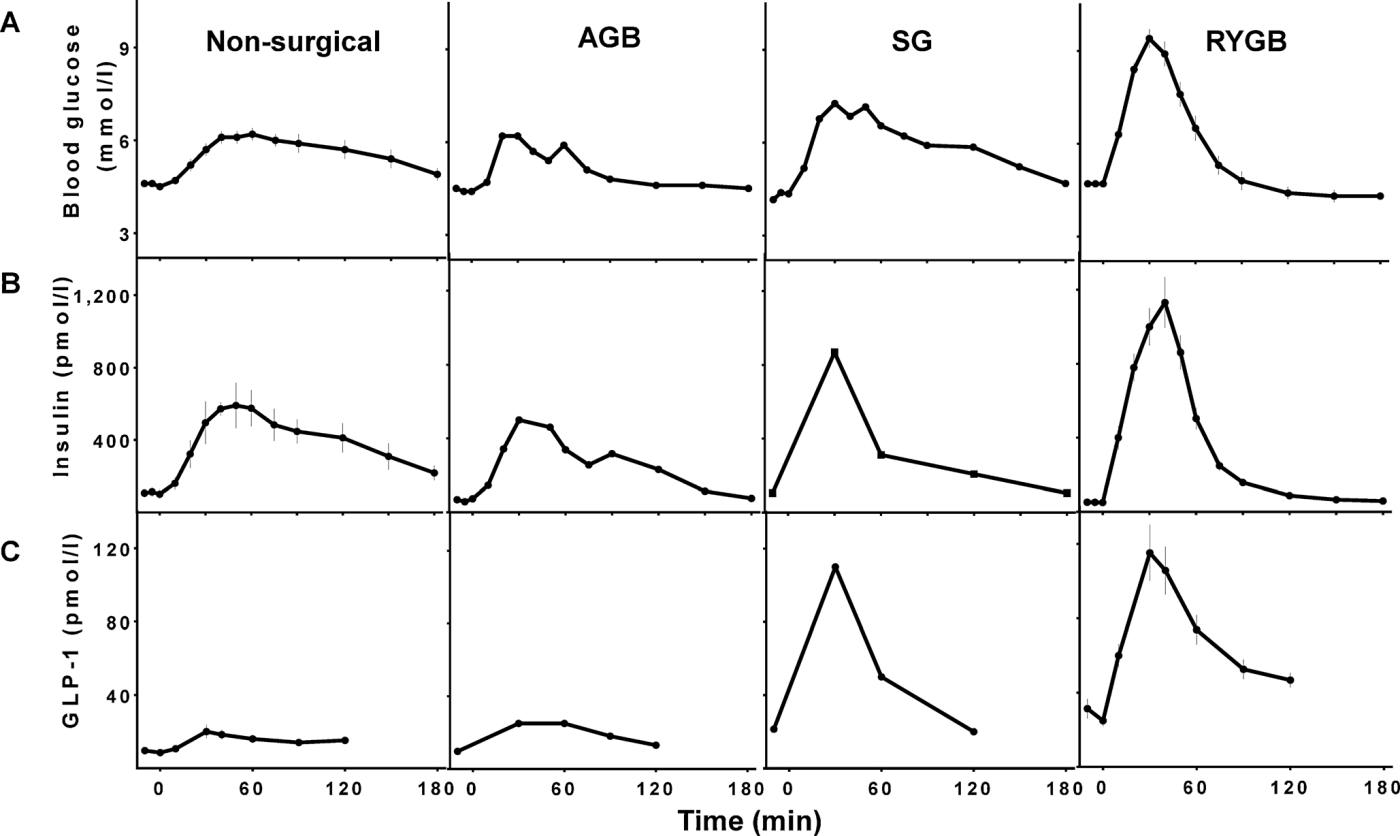
Blood glucose (**A**), insulin (**B**), and GLP-1 (**C**) response to liquid meal or oral glucose ingestion in non-surgical healthy controls and those after AGB, SG, and RYGB [29, 46, 51]. Data were adjusted for baseline values

After GB, and to lesser extent after SG, meal ingestion results in earlier and higher peaks of glucose as well as rapid declines to a lower glucose nadir. In parallel there is a similar pattern of insulin and GLP-1 secretion with earlier and larger peaks compared to people without surgery [20, 25, 27–30] (Fig. 1). This pattern is due in part to more rapid transit of nutrients into the small intestine from the restricted gastric compartments that inherent to these surgeries. Thus, there are large fluxes of nutrients onto the absorptive surface of the gut, and glucose into the splanchnic circulation [23, 24, 30]. Gastric emptying in individuals with an intact GI tract is tightly regulated [31, 32] due in part to feedback from small intestinal signals activated by luminal nutrients [31]. While gastric banding seems to have no effect on nutrient emptying [33], GB and SG are associated with an increased pace of nutrient passage through the gastric pouch/stomach into the small intestine [34, 35]. Emptying of the stomach pouch in individuals after GB is about 2–3 times faster than that in non-surgical healthy controls for both liquid and solid markers [36]. Following GB, rapid pouch emptying is attributed primarily to the size of the gastric outlet and the pressure gradient across the gastrojejunostomy [37], and is likely to be independent of neural or hormonal factors. Compared to non-surgical subjects, SG also leads to a 1.5–2 fold decrease in the half-time of gastric emptying, and increased small intestinal transit time for both liquid and solid food [38, 39], which is smaller than what has been reported after GB.

Given that procedures utilizing the foregut bypass have been described to improve glycemic control in non-obese [40–42] or mildly obese individuals [43], independent of weight loss, these procedures have been investigated as primary treatments for diabetes [44]. Glycemic improvement measured as the average A1C reduction at 6–21 months after some version of foregut bypass ranged from 2 to 4 % [40–42]. RYGB led to A1C reduction of 3 % at 1 y in 66 mildly obese patients with uncontrolled long lasting type 2 diabetes, and glycemic control was maintained up to 5 years, [43]. In these clinical studies, the amount of weight loss did not correlate with indices of glycemic improvement after surgery [41, 43].

**Fig. 2:**
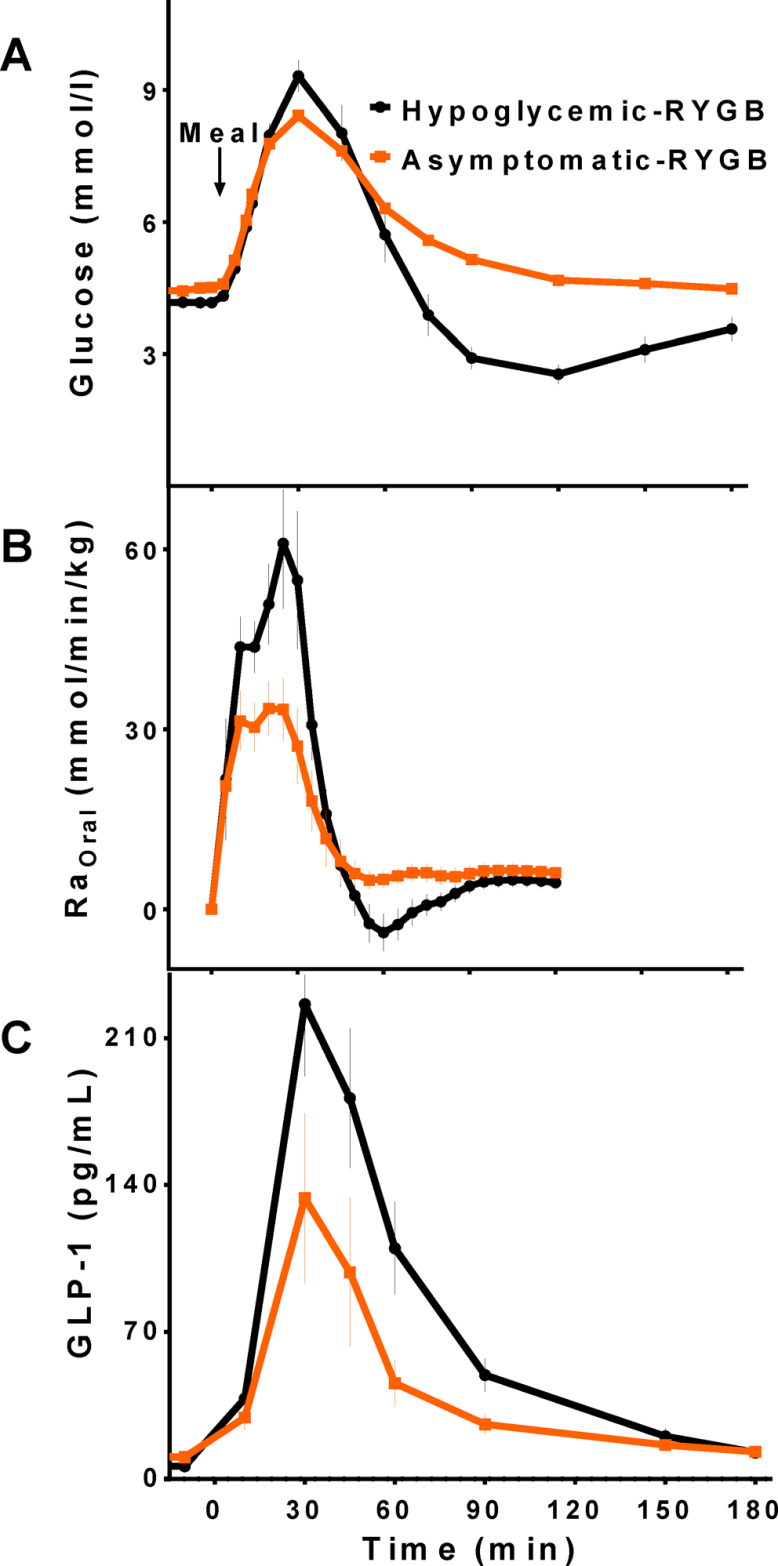
Blood glucose (**A**), systemic appearance of ingested glucose (Ra_Oral_) (**B**), and GLP-1 (**C**) levels during meal tolerance test in patients with hypoglycemia after RYGB and those without

The substantial impact of gastric bypass on glucose metabolism is exemplified by the syndrome of postprandial hyperinsulinemic hypoglycemia, which occurs in a small subset of subjects, but has not been reported after restrictive bariatric procedures or SG. Affected patients have larger meal-derived glucose appearance along with larger insulin and GLP-1 secretion compared to the background population who have undergone RYGB [45] (Fig. 2). The exaggerated insulin response in these individuals is not significantly related to the amount of weight loss or insulin sensitivity [46].

## Enteroinsular activity after bariatric surgeries

It has been recognized that postprandial glucose metabolism and islet function are tightly regulated by gut factors, collectively termed incretins. The greater insulin secretion in response to oral compared to intravenous (IV) glucose administration is termed the incretin effect and provides a quantitative measure of the effects of insulinotropic factors initiated by meal ingestion/absorption. While the hormonal component of the enteroinsular axis, principally the two gut hormones, glucose-dependent insulinotropic polypeptide (GIP) and glucagon-like peptide 1 (GLP-1), is better understood, the incretin effect also contains direct nutrient and neural stimulation [47].

An improved beta-cell response to meal ingestion has been reported as early as 1 week after RYGB [20]. Moreover, this effect is specific to enteral nutrients, since the insulin response to intravenous glucose, which has no effect on the incretin effect, is not changed by surgery [26, 48, 49]. One possibility that is frequently raised to explain the augmentation of insulin secretion after RYGB, and potentially the weight-loss independent improvement in glucose metabolism and diabetes, is increased action of the incretins. In this model, delivery of nutrients directly into the intestine leads to an increased secretion of insulinotropic hormones from the GI tract that parallels the higher rates of nutrient flux after RYGB. In fact, secretion of both incretins is changed by surgery. GIP levels shift upwards and to the left, consistent with a more rapid response to eating, although the 2–3 h integrated values of GIP are no different from individuals without surgery [46]. However, GLP-1 secretion after RYGB and SG is substantially enhanced. Both early and overall GLP-1 responses to test meals are higher, sometimes as much as 10-fold, those of un-operated controls or subjects with gastric restrictive surgery [23, 45, 50–52]. This effect can also be seen in subjects studied before and after surgery [25, 53]. Compared to controls, SG also enhances GLP-1 secretion but to lesser extent than RYGB [14, 29] (Fig. 1).

GLP-1 is a product of the proglucagon gene that is synthesized by intestinal endocrine L-cells, located with greatest density in the ileum and colon. GLP-1 is secreted shortly after nutrient ingestion, and plasma levels increase 2–3 fold in healthy individuals [54]. Once released to the circulation, GLP-1 is rapidly metabolized by a widely distributed serine protease, dipeptidyl peptidase IV (DPP-4) [55], such that the half-life of bioactive GLP-1 is less than 2 min in humans. Because of the drainage of the gut, concentrations of GLP-1 in the hepatic portal vein are approximately twice those in peripheral circulation [56]. GLP-1 actions are mediated through a receptor specific for GLP-1 (GLP-1r), expressed in different tissues, including pancreatic islet cells, some brain areas (hypothalamus, hindbrain and midbrain), portal vagal afferent nerves, gastric mucosal cells, lung, heart and kidney. Exogenous administration of GLP-1 improves glucose tolerance by increasing insulin secretion [57], suppressing glucagon secretion [58], delaying gastric emptying [59], decreasing endogenous glucose production [60].

While the underlying mechanisms by which gastric bypass surgeries or SG enhance the GLP-1 response is not totally understood, the rapid passage of nutrients into the intestine could partially account for this effect [61, 62]. However, postprandial hyperinsulinemia after GB and SG can likely be attributed to the combined effects of elevated glucose and GLP-1. RYGB has shown to increase incretin effect [25] independent of weight loss while the magnitude of the incretin effect after SG has not been clearly defined.

## The role of GLP-1 on glucose metabolism after bariatric surgeries

The elevated concentrations of GLP-1 that are a consistent and persistent effect of RYGB [63] has led to considerable speculation that this glucoregulatory peptide could have a central role in post-surgical effects. Recent work in humans supports this proposal. However, there are now several preclinical studies using mouse models with GLP-1 receptor deletions that raise questions as to the necessity of GLP-1 signaling after bariatric surgery. There have been great advances in the development of surgical models in rodents and both RYGB and SG can be replicated with fidelity in small animals, including reliable weight loss, improved glucose tolerance and increased plasma GLP-1. GLP-1 receptor knockout mice lost equivalent weight and had comparable glucose tolerance to control animals after RYGB [64] and SG [65]. While these studies have the strength of a well-defined molecular mechanism to isolate the effect of GLP-1 signaling, there are several caveats that should be considered when extending the results to humans. However, animals with developmental gene deletions and lifelong absence of GLP-1 signaling may develop compensations that allow them to have apparent normal function. Thus, the disparate results in humans and mice on the role of GLP-1 to mediate effects of bariatric surgery require more study for a definitive conclusion.

The physiologic actions of endogenous GLP-1 on glucose metabolism have been studied using continuous infusion of a potent GLP-1r antagonist, exendin-[9–39], Ex-9. Post-meal glucose homeostasis is tightly regulated as a result of GLP-1 action since elimination of this action causes a deterioration of glucose tolerance [66]. However, interpretation of the effect of GLP-1r blockade on insulin response during meal or oral glucose studies is complicated due to increased glycemia as a result of Ex-9 infusion. Studies with glucose or meal ingestions during fixed blood glucose concentrations with a hyperglycemic clamp has demonstrated that blocking endogenous GLP-1 reduces postprandial insulin secretion by 30-40 % and increases glucagon release [67]. These findings indicate that endogenous GLP-1 has a significant insulinotropic effect in healthy humans, and important glucagonostatic properties as well. Of note, the relative contribution of the GLP-1 effect to postprandial insulin secretion is similar in patients with well-controlled T2DM and age- and weight-matched controls [68], despite an absolute reduction in beta-cell function in the diabetic individuals. The insulintropic effect of GLP-1 does not seem to be mediated through alteration in gastric emptying since endogenous GLP-1, unlike pharmacological levels of GLP-1 or GLP-1 agonists, has only modest effects on the rate of nutrient passage from the stomach to the small intestine [67–69].

**Fig. 3:**
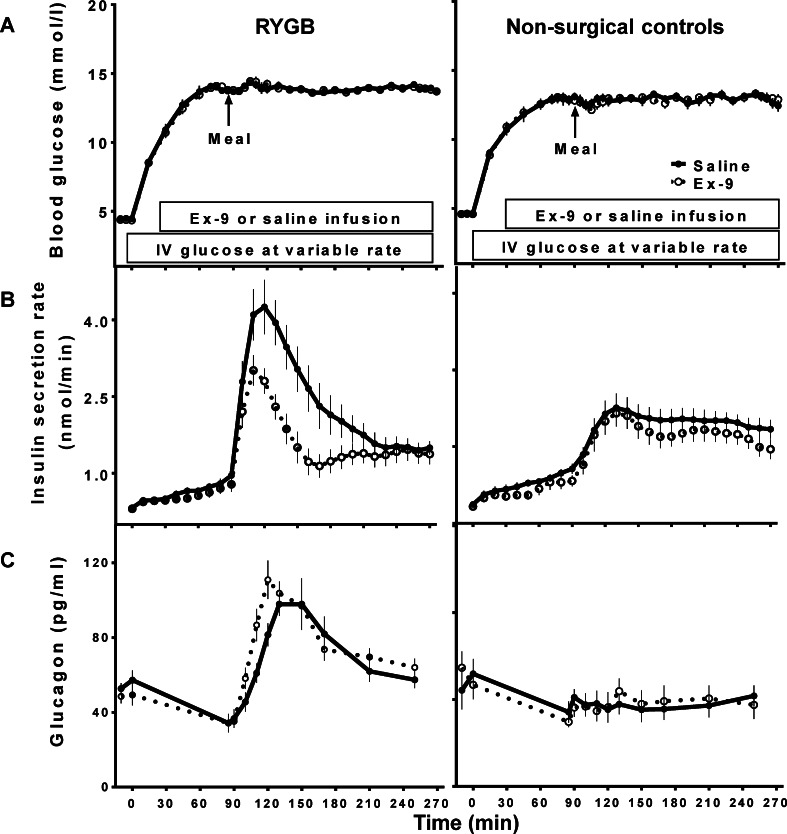
Blood glucose (**A**), insulin secretion rates (**B**), and glucagon (**C**) before and after meal ingestion during hyperglycemic clamp studies with (*dashed line*) and without (*solid line*) GLP-1r blockade

The effect of GLP-1 on insulin secretion after gastric bypass surgery was examined using Ex-9 infusion during a liquid mixed-meal tolerance study while blood glucose levels were maintained at 240 mg/dl in non-diabetic individuals with and without history of gastric bypass surgery [26] (Fig. 3). Surgical subjects had greater insulin secretion to nutrient ingestion compared to the controls and this effect was partly attributed to exaggerated endogenous GLP-1 action in these individuals since Ex-9 reduced insulin secretion by 33 ± 4 % in the RYGB group compared to 16 ± 5 % in the controls. Blocking GLP-1r resulted in larger glucagon response to meal ingestion in both surgical and non-surgical subjects, suggesting that the glucagonostatic effect of GLP-1 is also preserved after RYGB. Systemic appearance of ingested d-xylose was not influenced by GLP-1r blockade in either group, demonstrating that nutrient passage from the stomach/gastric pouch to small intestine is not affected as a result of ex-9 during a hyperglycemic clamp. This was the first study to address the direct role of endogenous GLP-1 on islet function in RYGB and the results confirmed the hypothesis that the greater circulating GLP-1 levels after surgery lead to greater GLP-1 action. Extension of these experiments to meal studies confirmed these findings, with RYGB subjects showing a larger effect of GLP-1r antagonism on glycemic excursion than non-operated controls (change in AUC_Glucose(3hr)_: 37 ± 12 % *versus* 14 ± 12 % [45]). In this study, where postprandial glucose was not clamped, Ex-9 shortened the time to reach the peak of oral glucose appearance in both groups, indicating more rapid nutrient passage as a result of blocking GLP-1 action. In keeping with these findings, other investigators have reported a larger increase in the postprandial glycemia in surgical subjects compared to non-operated controls (change in AUC_Glucose(6hr)_: ~45 % *vs.* ~24 %) as a result of Ex-9 infusion [45]. Blocking GLP-1r in this study increased the rate of radiolabeled meal transit in RYGB subjects indicating that GLP-1 regulates the emptying of the gastric pouch after surgery.

The role of enhanced GLP-1 action in the antidiabetic effects of RYGB was investigated by two independent groups using different methods. In the first study [70], 9 patients with well controlled diabetes consumed liquid mixed-meals with and without infusion of Ex-9 before surgery, and 1 week and 3 months after surgery. Eight of these subjects were taking antidiabetic medications (metformin alone or in combination with insulin secretagogues or insulin) before surgery, but none required any medication after surgery with normal HbA1c levels by 3 months. After surgery the subjects had earlier and larger glycemic excursions, with left-shifted insulin responses and increased β-cell glucose sensitivity. Ex-9 infusion increased glucose levels both before and after surgery with a 30 % increase in the 4 h glycemic response. Blocking GLP-1r diminished the insulin secretion to levels similar to those before surgery, indicating that a major portion of the glycemic benefit of RYGB is attributable to the insulinotropic effect of endogenous GLP-1. Similar to previous studies, Ex-9 increased glucagon and the beneficial effects of GLP-1 action was not dependent on changes in gastric emptying. A second study compared 8 patients with T2DM resolved after RYGB, and 7 age-matched and leaner non-diabetic controls, using Ex-9 to block GLP-1r during a liquid test meal [71]. The overall glucose response (AUC_Glucose(2hr)_) to the test meal was greater in the surgical subjects, as were their insulin responses. Ex-9 increased post-meal glycemia similarly in both groups similarly, ~10 %, but with greater suppression of insulin secretion in the surgical subjects. Taken together these findings demonstrate that the contribution of endogenous GLP-1 to postprandial insulin secretion is enhanced after RYGB, and improvement in postprandial glucose metabolism after surgery is at least partly due to this effect. By extension, it is plausible that enhanced GLP-1-stimulated insulin secretion is even further exaggerated in individuals who suffer from postprandial hyperinsulinemic hypoglycemia. Blocking GLP-1r corrected hypoglycemia in a group of subjects with GB-related postprandial neuroglycopenic hypoglycemia [45], and the contribution of GLP-1 to postprandial insulin secretion was larger in affected individuals compared to asymptomatic RYGB subjects. This finding is consistent with a significant role for GLP-1 action in pathogenesis of post-GB hypoglyemia. In this cohort, subjects with hypoglyemia after GB also had larger meal-derived glucose appearance compared to those without hypoglycemia, implying that increased nutrient flux contributes to enhanced GLP-1 action. While elimination of GLP-1 action has a larger effect on glycemia among subjects after RYGB, which is accentuated in those with the hyperinsulinemic hypoglycemia syndrome, it is not clear whether the increased GLP-1 action is due to larger GLP-1 secretion or greater beta-cell responsiveness to GLP-1.

The level of expression of islet GLP-1 receptors in pancreatic tissue samples obtained from 6 subjects with RYGB-related hypoglycemia did not differ from non-surgical controls [72]. Moreover, we have found that β-cell sensitivity to a step-wise incremental infusions of GLP-1 or GIP are not increased in non-diabetic subjects after GB compared to BMI- and age- matched non-surgical controls with normal glucose tolerance (M Salehi, unpublished). Based on these preliminary findings, enhanced GLP-1 action after GB does not seem to be due to increased beta-cell responsiveness to GLP-1. However, circulating levels of GLP-1 do not seem to entirely account for the high level of GLP-1 action in bariatric subjects since plasma GLP-1 concentrations do not correlate with the GLP-1-induced insulin response [26]. The portal-systemic gradient in glucose and GLP-1 levels has been previously reported [56]; it is possible that the gradient is larger after reconfiguration of GI tract and variable among surgical individuals given the anatomical differences.

The role of the enteroinsular axis in postprandial glucose homeostasis has not been studied in human after SG but SG appears to have similar effects on glucose, insulin, and GLP-1 responses to meal ingestion as GB [29], only smaller [14].

In summary, the bulk of experimental data from humans support a role for increased GLP-1 secretion and action to account for the characteristic patterns of insulin secretion and glucose regulation seen after RYGB and possibly SG. It is important to note that there is virtually no data from human studies to implicate or rule out a role for increased GLP-1 in the anorectic and weight loss effects of bariatric surgery. Improvements in assays for plasma GLP-1 and the availability of a well-defined GLP-1 receptor agonist will allow more refined hypotheses, such as the role of inter-individual variability in sensitivity to GLP-1, to be explored in bariatric patients. This area of research is likely to provide a valuable foothold for extending the understanding of physiologic mechanisms in weight loss surgery and for development of novel therapeutic approaches for treatment of diabetes.
